# Study of pulmonary hypertension in post-COVID-19 patients by transthoracic echocardiography

**DOI:** 10.1186/s43168-023-00201-w

**Published:** 2023-06-01

**Authors:** Heba Abdelhady Taha, Basem Ibrahim Elshafey, Taimor Mostafa Abdullah, Heba Ahmed Salem

**Affiliations:** 1 Chest Diseases Department, Faculty of Medicine, Tanta University, Tanta, Egypt; 2 Cardiovascular department, Faculty of medicine, Tanta university, Tanta, Egypt

**Keywords:** Pulmonary hypertension, COVID-19

## Abstract

**Background:**

A devastating medical disorder, the coronavirus pandemic infection (COVID-19), produced by the coronavirus 2 (SARSCoV-2), is primarily characterized by severe pneumonia. Pulmonary hypertension (PH), which may cause right ventricular (RV) involvement and dysfunction, can occur as a result of lung parenchymal injury and disturbed pulmonary circulation. Transthoracic echocardiography (TTE) is a very reliable noninvasive approach to determining the severity of PH. Similar to that, thorax computer tomography (TCT) can effectively detect the severity of lung damage during the acute phase of a COVID-19 infection.

**Aims:**

The goal of this research is to examine PH and altered right ventricular function by TTE in post-COVID-19 cases.

**Patients and methods:**

This retrospective case–control study was conducted at Tanta Chest University Hospital, Tanta, Egypt. The study started from October 2021 to September 2022 on 50 post-COVID-19 cases with one or more clinical manifestations of PH. These cases underwent TTE (group I showed normal PAP “control group,” groups II & III with PH further subdivided according to PaO_2_).

**Results:**

Risk factors of age, BMI, diabetes mellitus, and smoking were substantially raised in group III, but sex and hypertension were insignificant. Symptoms of chest pain, dyspnea, and palpitation were worse in group III. Levels of LDH, d-dimer, ESR, and serum bilirubin were substantially increased in group III in comparison to the other groups. Post-COVID-19-associated lung fibrosis and embolism were higher in group III. Mean values of estimated systolic pulmonary artery pressure (esPAP) and right atrial and right ventricular diameters were substantially increased in groups III and II in comparison to group I. Mean values of RV-GLS and TAPSE were lower in groups III and II in comparison to group I. FEV1, FVC, PEFR, and FEF_25–75%_ percentage of the predicted were significantly low in groups II and III. FEV1/FVC ratio was substantially lower in group II in comparison to groups I and III.

**Conclusions:**

The incidence of pulmonary hypertension in post-COVID-19 patients with suspected manifestations of PH is 70%. Increased age, BMI, DM, smoking, decreased PaO_2_, increased CORADS score, and abnormal spirometry are risk factors for PH in post-COVID-19 patients. Patients with post-COVID-19 PH stay more either in ICU or ward.

## Introduction

A new severe acute respiratory syndrome (SARS) coronavirus 2 (SARS-CoV-2) with the name COVID-19 was first discovered in Wuhan, China, in December 2019. The virus spreads quickly in other Chinese regions and causes atypical pneumonia in certain individuals before progressing to severe injury to the lungs and acute respiratory distress syndrome (ARDS). The remaining countries of the globe quickly were impacted, and on March 11, 2020, WHO declared COVID-19 to be a pandemic.

Egypt was verified to have caught the virus on February 14, 2020. The COVID-19 virus spreads by air droplet infection and tiny particles. People are infectious for up to 20 days and may transfer the infection even if they are asymptomatic [1].

The COVID-19 pandemic brought on by the SARS-CoV-2 virus has been causing devastation around the globe for over 2 years. The virus primarily affects the respiratory system, but it may exist in other organs with affinity, including the endothelium, neural tissue, myocardiocytes, and the gut, wherever the viral invasion was enabled by the presence of functioning ACE-2 receptors. The clinical course of COVID-19 may range from being completely asymptomatic to having severe respiratory distress syndrome that needs mechanical ventilation and is very fatal [2].

Since the COVID-19 outbreak, there have been a rising number of COVID-19 survivors who have struggled with the illness’ symptoms despite having received a clinically negative test. Long-haulers are the name given to them. The difficult part of managing COVID-19 sequalae as we fight this pandemic is that it can range from mild in terms of body aches and fatigue to severe forms requiring long-term oxygen therapy and transplantation of the lungs due to lung fibrosis, significant cardiac abnormalities, and stroke, all of which significantly impair quality of life. Numerous studies have shown that even after being deemed COVID-free, 70–80% of people who recovered from COVID-19 still exhibit at least one or more symptoms.

Right ventricular dysfunction (RVD) and pulmonary hypertension (PH) are one of the controversial complications of COVID-19 either secondary to post-COVID-19 lung fibrosis or pulmonary thromboembolism, which will be covered in this study [3, 4].

A mean pulmonary arterial pressure of more than 20 mmHg at rest is currently the criteria of pulmonary hypertension (PH). A pulmonary vascular resistance (PVR) > 2 Wood units and a pulmonary arterial wedge pressure ≤ 15 mmHg are also implied by the definition of pulmonary arterial hypertension (PAH).

The pulmonary vessels are constricted, obstructed, or damaged in pulmonary arterial hypertension (PAH). Due to the injury, blood pressure in the pulmonary artery increases, and blood flow to the lungs slows down. Blood must be pumped through the lungs more forcefully by the heart. The extra effort on the cardiac muscles leads to its weakness causing right ventricular dysfunction [5].

Pulmonary hypertension worsens gradually in some persons and may be fatal. Some forms of pulmonary hypertension have no known cure; however, therapy may help manage symptoms and enhance quality of life [5, 6].

## Patients and methods

Retrospective case–control research was done in this study at Tanta Chest University Hospital, Tanta, Egypt, From October 2021 to September 2022 on 50 post-Covid-19 patients with one or more clinical manifestations of pulmonary hypertension.

### Inclusion criteria

- Real-time reverse transcriptase-polymerase chain reaction (RT-PCR) testing of pharyngeal and nasal swabs revealed positive results in patients with COVID-19.
- Fifteen days must pass from the start of symptoms, with the last 3 days fever-free, until 3 months with 2 RT-PCR negative for COVID-19 with one or more clinical manifestations of PH or right ventricular failure (RVF), and without preexisting cardiovascular conditions.

### Exclusion criteria

- Participants with cardiovascular disorders, especially pulmonary hypertension
- Patients with a history of chronic chest diseases
- Patients with rheumatological diseases that may cause pulmonary hypertension

Echocardiography was done to all patients, 15 patients with mean age of 53.27 ± 13.38 (8 males and 7 females) showed normal pulmonary artery pressure “group I,” and 35 participants had criteria of increased pulmonary artery pressure (PAP) and were classified according to PaO_2_ to 2 groups:

- *Group II*: Comprised 15 participants with a mean age of 52.07 ± 14.28 (6 males and 9 females) with PaO_2_ more than or equal to 60 mmHg on room air at sea level
- *Group III*: Comprised 20 participants with a mean age of 58.8 ± 14.68 (12 males and 8 females) with PaO_2_ less than 60 mmHg on room air at sea level

The following procedures were applied to all patients:

- Thorough taking history epidemiological data and symptoms of pulmonary hypertension (chest pain, dyspnea, and palpitations)
- Clinical examination to confirm pulmonary hypertension and RVF
- Imaging (chest CT for parenchymal lung affection with T-CT CORADS scoring, CT chest with pulmonary angiography for diagnosis of pulmonary embolism was performed when suspected.)
- Laboratory investigations (ABG, D-dimer, LDH, serum bilirubin, ESR, PCR (RT-PCR) for COVID-19)
- Standard 12-lead ECG
- Resting transthoracic echocardiography (TTE) and measurement of the following:
  - Right atrial (RA) enlargement is indicated by a RA diameter in a 4-chamber view of more than 44 mm.
  - Right ventricular (RV) dilatation is indicated by a RV diameter of > 42 mm in a four-chamber view.
  - Based on the peak TRV, we determined that estimated systolic PAP (esPAP) values of ≥ 35 mm Hg at rest indicate PH with intensity varying from mild (35–44 mm Hg), up to moderate (45–60 mm Hg), whereas severe (> 60 mm Hg) [7, 8].
  - Values below 17 mm, measured using M-mode at the lateral tricuspid valve annulus, were deemed indicative of right ventricular dysfunction, specifically tricuspid annular plane systolic excursion (TAPSE).
  - Right ventricular dysfunction (RVD) was defined as either TAPSE under 17 mm and/or right ventricular global longitudinal strain (RV-GLS) under − 28% (borderline values for TAPSE were 17–20 and for RV-GLS − 25 to − 27). RV-GLS was measured in an apical four-chamber view [9].
- Pulmonary function test by spirometry and the following parameters were measured as a percent of predicted: Forced expiratory volume in 1 s (*FEV*_1_), forced vital capacity (FVC), *FEV*_1_/FVC, forced mid-expiratory flow (FEF_25-75%_), and peak expiratory flow rate (PEFR).

### Ethical considerations

The Tanta Chest University Hospital’s Ethics Committee, in Tanta, Egypt, gave its approval to the research. The following precautions are sufficient to protect participants’ privacy and the data’s confidentiality:

- The patients were given the option of not participate in the study if they did not want to.
- Each participant receives a code number, and their name and address are maintained in a separate file.
- When we utilize the research, we hide the patients’ names.
- The study’s findings were exclusively used scientifically; they were not utilized for any other purposes.

### Statistical analysis

SPSS v27 (IBM, Armonk, NY, USA) was used for the statistical analysis. Histograms and the Shapiro-Wilks test were used to assess the normality of the data distribution. The *F*-test (ANOVA test) was used to analyze quantitative parametric data that were reported as mean and standard deviation (SD). When applicable, qualitative variables were analyzed using the chi-square test or Fisher’s exact test and provided as frequency and percentage (%). Statistics were deemed to be significant at a two-tailed *P*-value < 0.05.

## Results

Among 50 post-COVID-19 patients, 30% of patients had normal pulmonary artery pressure, 30% had pulmonary hypertension with PaO_2_ ≥ 60 mmHg, and 40% had PH and RVD with PaO_2_ < 60 mmHg (Table 1).

**Table 1:** Pulmonary hypertension incidence in post-COVID-19 patients

Pulmonary hypertension (PHT) incidence in post COVID-19	*N*	%
No PHT	15	30
PHT with PaO_2_ ≥ 60	15	30
PHT with PaO_2_ < 60	20	40
Total	50	100

S1 wave, Q3 wave, and T3 wave were substantially varied among the 3 groups. *Deep* S1 wave and Q3 wave were much greater in group III than in groups I and II with *P*-value < 0.05, and groups I and II did not significantly vary from one another (*P* > 0.05). T3 *inverted* wave was much greater in group III than in groups I and II with *P*-value < 0.05, and groups I and II did not significantly vary from one another (*P* > 0.05) (Table 2).

**Table 2:** Percentage and statistical analysis of some ECG findings among the 3 groups

			**Group I**	**Group II**	**Group III**	*χ*^**2**^	***p*-value**
**S1 wave**	**Normal**	***N***	15	13	13	7.430	0.024*
		**%**	100.0%	86.7%	65.0%		
	**Deep**	***N***	0	2	7		
		**%**	0.0%	13.3%	35.0%		
**Q3 wave**	**Normal**	***N***	15	13	11	11.053	0.004*
		**%**	100.0%	86.7%	55.0%		
	**Dee**	***N***	0	2	9		
		**%**	0.0%	13.3%	45.0%		
**T3 wave**	**Normal**	***N***	15	14	12	11.156	0.004*
		**%**	100.0%	93.3%	60.0%		
	**Inverted**	***N***	0	1	8		
		**%**	0.0%	6.7%	40.0%		

^*^Significant as *p*-value ≤ 0.05

As regards *echocardiogram* in studied groups, it showed the following:

The mean value of *pulmonary artery pressure* (PAP) in mmHg was significantly increased compared with groups I and II in group III (*P*-value < 0.05), and PAP’s mean value also increased in group II in comparison to group I (*P*-value < 0.05). The mean value of *RV diameter and RA diameter* in (mm) showed a significant difference among the three groups as RV dilatation in groups II and III was considerably larger as compared to group I (*P*-value < 0.05), and there was an insignificant difference between groups II and III (*P* > 0.05). The mean value of *RV-GLS* (%) was markedly lower in group III in comparison to groups I and II (*P*-value < 0.05), and there was a marked decrease in group II in comparison to group I (*P* < 0.05). TAPSE’s mean value in (mm) was markedly lower in groups II and III in comparison to group I with (*P*-value < 0.05), and it was lower in group II in comparison to group I (*P*-value < 0.05) (Table 3).

**Table 3:** Range, mean, standard deviation (SD), and statistical analysis of some echocardiographic measurements in the three studied groups

		Range			Mean	±	SD	*F*-test	*p*-value		
Pulmonary artery pressure (mmHg)	Group I	15	–	30	22.80	±	4.26	72.979	0.001*	P1	0.001*
	Group II	35	–	65	47.07	±	11.49			P2	0.001*
	Group III	40	–	85	65.60	±	12.47			P3	0.001*
Right ventricle diameter (mm)	Group I	28	–	41	34.93	±	4.37	10.050	0.001*	P1	0.001*
	Group II	32	–	58	42.67	±	7.58			P2	0.001*
	Group III	32	–	56	43.65	±	5.79			P3	0.635
Rt atrial diameter (mm)	Group I	28	–	44	34.20	±	4.54	13.596	0.001*	P1	0.001*
	Group II	33	–	55	41.67	±	7.41			P2	0.001*
	Group III	34	–	55	44.05	±	4.82			P3	0.223
RV-GLS (%)	Group I	− 50	–	− 32	− 38.27	±	5.33	87.093	0.001*	P1	0.001*
	Group II	− 40	–	− 17	− 25.07	±	6.38			P2	0.001*
	Group III	− 21	–	− 10	− 15.7	±	3.34			P3	0.001*
TAPSE (mm)	Group I	2.3	–	4.6	3.47	±	0.82	44.776	0.001*	P1	0.001*
	Group II	1.4	–	4.1	2.46	±	0.83			P2	0.001*
	Group III	1.2	–	1.6	1.41	±	0.13			P3	0.001*

^*^Significant as *p*-value ≤ 0.05 (*RV-GLS* right ventricular global longitudinal strain, *TAPSE* tricuspid annular plane systolic excursion)

The percentage of patients’ number diagnosed with post-COVID-19 *pulmonary fibrosis* was much greater in group I than in groups II and III, and compared to group III, it was statistically highly significant in group II, with *P*-value < 0.05). The percentage of patients’ number diagnosed with post-COVID-19 *pulmonary fibrosis in association with pulmonary embolism* was markedly greater in group III as compared to groups I and II, and it was greater in group II in comparison to group I with *P*-value < 0.05) (Table 4).

**Table 4:** Percentage and statistical analysis of some radiological data in the 3 studied groups

			Group I	Group II	Group III	*χ*^2^	*p*-value
Radiological data	Fibrosis	*N*	15	13	10	13.085	0.001*
		%	100.0%	86.7%	50.0%		
	Fibrosis + embolism	*N*	0	2	10		
		%	0.0%	13.3%	50.0%		

^*^Significant as *p*-value ≤ 0.05

*LDH* mean values (U/L) and *D-dimer* level in mg/FEU were markedly greater in group III in comparison to group I with (*P*-value < 0.05), and they were insignificant between groups I & II with *(P*-value > 0.05). *ESR* mean values in (mm/h) and *serum bilirubin* in (mg/dL) were markedly increased in group III in comparison to groups I and II with *P*-value < 0.05, and they were significantly increased in group II in comparison to group I (*P* < 0.05). Regarding arterial blood gases (ABG), it was found that mean values of *PO*_*2*_ (mmHg) and oxygen saturation (*SO*_*2*_) % were markedly reduced in group III in comparison to groups I and II (*P*-value < 0.05), and they were lower in group II in comparison to group I (*P* < 0.05). PH, PCO_2_, and HCO_3_ were insignificantly different among the three groups (Table 5).

**Table 5:** Range, mean, standard deviation (SD), and statistical analysis of LDH, ESR, S. bilirubin, D-dimer, and ABG in the three studied groups

		Range			Mean	±	SD	*F*-test	*p*-value		
LDH (U/L)	Group I	110	–	652	309.00	±	188.54	21.340	0.001*	P1	0.790
	Group II	150	–	477	291.07	±	97.61			P2	0.001*
	Group III	190	–	985	644.80	±	222.70			P3	0.001*
ESR (mm/hour)	Group I	10	–	110	35.20	±	29.75	18.705	0.001*	P1	0.002*
	Group II	15	–	120	70.00	±	34.43			P2	0.001*
	Group III	25	–	130	97.25	±	25.62			P3	0.010*
Serum bilirubin (mg/dL)	Group I	0.2	–	1.1	0.59	±	0.30	15.183	0.001*	P1	0.003*
	Group II	0.2	–	1.8	0.99	±	0.38			P2	0.001*
	Group III	0.6	–	2	1.25	±	0.37			P3	0.034*
D-dimer (mg/ FEU)	Group I	0.2	–	1.5	0.59	±	0.44	8.153	0.003*	P1	0.093
	Group II	0.4	–	2.6	1.61	±	0.81			P2	0.001*
	Group III	0.3	–	9.1	2.82	±	2.43			P3	0.035*
PO_2_ (mmHg)	Group I	60	–	98	76.40	±	11.62	86.408	0.001*	P1	0.009*
	Group II	60	–	80	68.42	±	5.88			P2	0.001*
	Group III	38	–	59	42.83	±	5.68			P3	0.001*
SO_2_%	Group I	93	–	98	95.87	±	1.41	72.088	0.001*	P1	0.011*
	Group II	83	–	98	91.45	±	4.79			P2	0.001*
	Group III	70.8	–	89	78.16	±	5.80			P3	0.001*
HCO_3_ (mEq/L)	Group I	12	–	28	24.23	±	4.59	0.341	0.713	P1	0.451
	Group II	22	–	29	25.40	±	2.50			P2	0.894
	Group III	12	–	29	24.43	±	4.86			P3	0.501
PH	Group I	7.3	–	7.6	7.43	±	0.09	0.815	0.449	P1	0.373
	Group II	7.42	–	7.53	7.47	±	0.03			P2	0.219
	Group III	7	–	7.7	7.48	±	0.15			P3	0.777
PCO_2_ (mmHg)	Group I	18	–	40	32.07	±	6.79	0.245	0.784	P1	0.841
	Group II	26	–	42	32.67	±	5.25			P2	0.649
	Group III	15	–	60	30.80	±	10.44			P3	0.503

^*^Significant as *p*-value ≤ 0.05 (*LDH* lactate dehydrogenase, *ESR* erythrocyte sedimentation rate)

The mean values of *durations of ICU stay and total hospital stay* (calculated as the sum up of patients’ duration of stay in ICU and ward) in days were significantly longer in group III as compared to groups I and II with *P*-value < 0.05, while there was no significant difference between groups I and II with *P* > 0.05 (Table 6).

**Table 6:** Range, mean, standard deviation (SD), and statistical analysis of duration of hospital stay between ICU and total hospital stay in the three studied groups

		Range			Mean	±	SD	*F*-test	*p*-value		
**ICU stay (days)**	**Group I**	0	–	3	0.33	±	0.90	61.030	0.001*	P1	0.223
	**Group II**	0	–	5	1.13	±	1.77			P2	0.001*
	**Group III**	2	–	10	6.35	±	2.21			P3	0.001*
**Hospital total stay (days)**	**Group I**	6	–	12	8.20	±	1.82	40.918	0.001*	P1	0.099
	**Group II**	7	–	16	9.93	±	2.76			P2	0.001*
	**Group III**	10	–	21	16.30	±	3.40			P3	0.001*

^*^Significant as *p*-value ≤ 0.05

## Discussion

In this study, the incidence of pulmonary hypertension was proportionate to the severity of cases as among 50 post-COVID-19 patients, 30% of patients had normal pulmonary artery pressure, 30% had pulmonary hypertension with PaO_2_ ≥ 60 mmHg, and 40% had PH and RVD with *PaO*_*2*_ < 60 mmHg. The possible mechanism of elevated pulmonary artery pressure, RV dysfunction, and dilation are probably multifactorial. Some of these processes include hypoxic vasoconstriction, thrombotic events, direct viral damage, proinflammatory cytokines, and most likely increased afterload and overload [10]. High D-dimer assessments might indicate that COVID-19 patients have a greater level of blood coagulation cascade activation as a result of a systemic inflammatory responses syndrome or as a direct outcome of the SARS COV-2. Hyperbilirubinemia could be noted in this study in severe cases, The viral infection of the hepatocytes by SARS-COV-2 may result in hepatic injury due to the well-expressed ACE-2 receptors in hepatic cells and bile duct epithelium or by the medications used. Increased LDH levels are directly proportionate with the severity of the case and organ failure as it has five isoenzymes in the human body that are presented in cardiomyocytes and pneumocytes, so it shows higher levels in severe cases of pulmonary hypertension, right ventricular dysfunction, and respiratory failure. Sustained high ESR levels after recovery from COVID-19 infection might be triggered by the virus itself in the acute stage that leads to changes in the characteristics of the erythrocytes or plasma including the immune system by unknown mechanism, so it has prognostic value in post-COVID-19 infection. The duration of patients’ total hospital stay (calculated as a sum up of patients’ duration of stay in ICU and ward) in days is directly proportionate to the severity of the condition as in group III most cases needed long period of ICU stay for improving respiratory distress and failure that needed high-flow oxygen therapy and some needed assisted ventilation.

In line with our research, *Qing Deng *et al*. (2020)* [11] performed a retrospective study and gathered clinical data related to the heart, particularly including heart imaging results, laboratory findings, and clinical outcomes and discovered that 13.4% of individuals had PH symptoms but were classified as low probability (peak tricuspid regurgitation velocity < 2.8 m/s or absence of at least two echocardiographic signs).

Moreover, *Pagnesi *et al*. (2020)* [12] study included 211 COVID-19 participants; they were enrolled in a single-center, observational, cross-sectional research. At the transthoracic echocardiography (TTE) evaluation, patients were divided into groups based on the existence or the absence of RVD and PH. They discovered that the incidence of RVD and PH was, respectively, 12.0% (24/200) and 14.5% (29/200).

*Barman *et al*. (2021)* [13] investigated the link between electrocardiographic results and the markers of the intensity of COVID-19 observed on electrocardiography (ECG) in a study on a number of 219 participants who were hospitalized due to COVID-19. According to the degree of the COVID-19 infection, patients were divided into two groups: non-severe and severe. They discovered that the severe group observed deep S1 waves, Q3 waves, and T3 waves more frequently than the non-severe group.

*Erdem and Duman (2023)* [14] found that sPAP in ICU patients was substantially greater than in COVID-19 ward patients or outpatients. Moreover, patients treated in the COVID-19 unit had considerably greater sPAP and mPAP levels than outpatients. Additionally, the ICU patients’ basal diameter, RV dimensions, longitudinal dimensions, and mid-diameter were substantially greater than those of the COVID-19 ward patients and outpatients. The COVID-19 ward patients’ RV diameters were similarly substantially greater than those of the outpatients. TAPSE was reduced in ICU patients compared to outpatients.

In agreement with this result, *Ozer *et al*. (2021)* [15] conducted a prospective, single-center research on 79 participants to analyze the right ventricular (RV) comprehensively using echocardiography in COVID-19 recovery patients. According to the intensity of pneumonia or its absence, the patients were separated into 4 groups: those with severe pneumonia, those with mild to moderate pneumonia, those without pneumonia, and the control group. When compared to the control group, they discovered that individuals with severe pneumonia had greater RV diameters. Compared to the control group, RV-GLS was diminished. PAP was greater in the groups with severe and mild-to-moderate pneumonia compared to the groups without pneumonia and controls. Subclinical RV systolic function impairment was found in hospitalised COVID-19 individuals and maintained after recovery in association with the severity of pneumonia.

Similarly, *Tudoran *et al*. (2021)* [16] showed that pulmonary artery pressure compared to the mild group was considerably greater in the moderate group. Also, TAPSE was substantially higher in the mild group compared to the moderate group.

Also, *Pagnesi *et al*. (2020)* [12] found that pulmonary artery pressure and RV diameter were substantially greater in the PH group than when the PH group was not present. Also, they observed that TAPSE was substantially lower in the group of people with pulmonary hypertension than in the absence of pulmonary hypertension group.

Similarly, *Léonard-Lorant *et al*. (2020)* [17] conducted a retrospective study on 160 participants. One-hundred six of these 160 individuals were identified as having COVID-19 infection (97 participants by RT-PCR and 9 participants with positive CT and negative RT-PCR test). They discovered that extremely sick COVID-19-infected individuals had a higher-than-normal risk of pulmonary embolism.

Moreover, *Grillet *et al*. (2020)* [18] performed a retrospective study on 100 individuals with severe clinical characteristics and COVID-19 infection to assess the relationship between pulmonary embolism and COVID-19 infection utilizing pulmonary CT angiography. They discovered that individuals with COVID-19 had a significant frequency of acute pulmonary embolism.

*Pagnesi *et al*. (2020)* [12] found that D-dimer and LDH were substantially greater in the pulmonary hypertension group than the absence of pulmonary hypertension group.

Similarly, *Léonard-Lorant *et al*. (2020)* [17] found that individuals with COVID-19 infection with pulmonary embolism had greater levels of D-dimer than patients without pulmonary embolism.

In agreement with this result, *Pagnesi *et al*. (2020)* [12] found that SpO2 was substantially reduced in the PH group in comparison to the absence of PH group.

Moreover, *Munker *et al*. (2022)* [19] demonstrated that the critical disease group PaO2 was substantially lower than the mild and moderate disease groups. However, PaCO2 was insignificantly different among the three groups.

Similarly, Tudoran et al. (2021) [16] showed that O2 saturation was substantially reduced in the moderate group in comparison to the mild group.

## Conclusion

In this study, the incidence of pulmonary hypertension in post-COVID-19 patients with suspected manifestations of pulmonary hypertension is 70%. Increased age, BMI, DM, smoking, decreased PaO_2_, increased CORADS score, and abnormal spirometry are risk factors for pulmonary hypertension in post-COVID-19 patients. Patients with post-COVID-19 pulmonary hypertension stay more either in ICU or ward.

### Limitations

The study was conducted on a small number of patients. This study depends on the study of echocardiography instead of the pulmonary catheter in the diagnosis of pulmonary hypertension.

## Data Availability

The author may be contacted for reasonable requests on the datasets utilized and/or analyzed in the present study.

## Abbreviations

ABG: Arterial blood gases
COVID-19: Coronavirus disease 2019
ACE-2: Angiotensin-converting enzyme 2
ESR: Erythrocyte sedimentation rate
esPAP: Estimated systolic pulmonary artery pressure
FEF_25–75%_: Forced mid-expiratory flow
FVC: Forced vital capacity
FEV1: Forced expiratory volume in 1 s
LDH: Lactate dehydrogenase
PEFR: Peak expiratory flow rate
PAP: Pulmonary artery pressure
RA: Right atrium
PH: Pulmonary hypertension
RV: Right ventricle
RVD: Right ventricular dysfunction
RT-PCR: Reverse transcription-polymerase chain reaction
SARS‐COV‐2: Severe acute respiratory syndrome coronavirus-2
RV-GLS: Right ventricular global longitudinal strain
TAPSE: Tricuspid annular plane systolic excursion
TTE: Transthoracic echocardiography
T-CT: Thoracic computed tomography
BMI: Body mass index
DM: Diabetes mellitus
PaO2: Arterial oxygen pressure
